# School-based comprehensive sexuality education for prevention of adolescent pregnancy: a scoping review

**DOI:** 10.1186/s12905-024-02963-x

**Published:** 2024-02-21

**Authors:** Su Mon Myat, Porjai Pattanittum, Jen Sothornwit, Chetta Ngamjarus, Siwanon Rattanakanokchai, Kyaw Lwin Show, Nampet Jampathong, Pisake Lumbiganon

**Affiliations:** 1 Department of Public Health, School Health Division, Ministry of Health, Naypyidaw, Myanmar; 2 Department of Epidemiology and Biostatistics, Faculty of Public Health, Khon Kaen University, Khon Kaen, Thailand; 3 Department of Obstetrics and Gynecology, Faculty of Medicine, Khon Kaen University, Khon Kaen, Thailand; 4 Department of Medical Research, Ministry of Health, Naypyidaw, Myanmar; 5 Cochrane Thailand, Khon Kaen University, Khon Kaen, Thailand

**Keywords:** School, Comprehensive sexuality education, Adolescent, Pregnancy, Scoping review

## Abstract

**Background:**

Adolescent pregnancy is a global public health problem. Numerous approaches for Comprehensive Sexuality Education (CSE) delivery in schools have been implemented around the world. Previous reviews on CSE did not follow the International Technical Guidance on Sexuality Education (ITGSE) because CSE is very diverse in terms of population, interventions, settings and outcomes. We conducted this scoping review to identify and map the evidence of school-based CSE for prevention of adolescent pregnancy with emphasis on adolescents’ contraceptive use, unintended pregnancy and abortion.

**Methods:**

We searched PubMed, CENTRAL, Scopus, ISI Web of Science, CINAHL, and WHO ICTRP to identify potential eligible studies from their inception to 4^th^ Nov 2023.We included randomized controlled trials (RCTs) and non-RCTs of CSE implemented in public or private schools for adolescents. CSE was defined as a multi-session intervention in school that covered topics including contraception, pregnancy, abortion, and HIV/STI. School-based interventions were the main intervention that may be either stand-alone or multicomponent. There was no limitation on study’s geographical area, but only English-language studies were considered. Two reviewers selected and extracted data independently, discussed for consensus or consulted the third reviewer if there were discrepancies for final conclusion. Data were presented using figures, map and table.

**Results:**

Out of 5897 records, 79 studies (101 reports) were included in this review. Most studies were conducted in the United States and other high-income countries in secondary or high schools with cluster RCTs. All studies included participatory methods. Almost all studies included Sexual and Reproductive Health which is the eighth concept of CSE. Very few studies reported the prespecified primary outcomes of contraceptive use during last sex, unintended pregnancy and abortion and hence this highlighted the gaps of available evidence for these outcomes. The number of concepts, components, duration and providers of CSE varied across the included studies. However, none of the interventions identified in this scoping review adhered to the ITGSE recommended approach.

**Conclusions:**

Our scoping review shows gaps in school-based CSE implementation in terms of completeness of concepts, components, providers, duration and outcomes recommended by ITGSE.

**Supplementary Information:**

The online version contains supplementary material available at 10.1186/s12905-024-02963-x.

## Background

Adolescent girls aged 15–19 years had an estimated 21 million pregnancies each year globally [1]. Half of these pregnancies were unintended and 55% of them ended in abortions [2, 3]. Pregnancy complications and unsafe abortions were the leading cause of death among adolescent girls [3]. About 14 million adolescents were not using contraception despite not wanting to become pregnant [2]. Only about one-third of adolescent girls in low- and middle-income countries (LMICs) used modern contraceptives, which consist of oral hormonal pills, injectables, male or female condoms, vaginal barrier methods, intrauterine devices, implants, female and male sterilization and emergency contraception [4].

Comprehensive Sexuality Education (CSE) was found to be effective in preventing and reducing unintended pregnancies in various countries [5].CSE is “a curriculum-based process of teaching and learning about the cognitive, emotional, physical, and social aspects of sexuality”. This is according to the 2018 revised edition of International Technical Guidance on Sexuality Education (ITGSE) by United Nations Educational, Scientific and Cultural Organization (UNESCO). Currently, the 2018 ITGSE (second edition) is perhaps the most comprehensive international guidance available for CSE. For delivering effective CSE, it must be integrated into existing curriculum or stand-alone subject and included multiple, sequential sessions over years [6].

CSE is one of the key interventions in adolescent-specific essential sexual and reproductive health and rights (SRHR) [7] which is recommended in several World Health Organization (WHO) guidelines [8–12].In addition, CSE is also one of the evidence-based health interventions for adolescents’ health described in Global Strategy for Women’s, Children’s and Adolescents’ Health (2016–2030) [13].

CSE can have positive effect on sexual behavior such as increase use of condom and contraception [14]. In 2016, a review on the effectiveness and implementation of CSE worldwide by UNESCO reported that CSE increased knowledge and improved attitudes related to SRH and contributed to the outcomes of increased condom and contraception use [6, 15]. Cochrane systematic reviews concluded that education alone was not effective and needed to be combined with contraceptive services [16, 17]. It was proven that CSE together with the policy and youth-friendly health services could increase adolescent contraceptive use to prevent pregnancy [18]. Moreover, the effect of CSE would be magnified if the laws, gender, poverty and social norms are addressed at a different level [7, 19].

Globally, 1.3 billion adolescents represent 16% of the global population [20] and 14% of all unsafe abortions in LMICs took place in adolescent girls [12]. In recent time, the unprecedented event of the US Supreme Court’s decision to overturn safe abortion services and comprehensive SRHR may have a huge impact and negative ripple effect on SRHR information and services in LMICs [21]. Some countries such as South Africa and Spain have access to legal abortion on request but such countries as in UK, Nigeria, Tanzania or Zambia, legal condition for abortion is to save the women lives and to protect her physical or mental health [22]. With the possibility of disrupted support and services, the need to access CSE for the current and future generations of adolescents is more pressing than ever [23].

Numerous approaches for CSE delivery in schools have been implemented around the world. Schools are the cost-effective and equitable platform for education and health sectors to work together to deliver essential adolescent SRHR interventions [7, 24]. Regarding the intervention, reviews prior to 2018 did not consider the recommended interventions according to ITGSE [16, 17, 25, 26]. CSE is very diverse in terms of population, interventions, settings and outcomes, we therefore conducted this scoping review to identify and map evidence on school-based CSE interventions to prevent adolescent pregnancy with emphasis on contraceptive use, unintended pregnancy, and abortion to improve adolescent SRHR and knowledge gaps in the contents and contexts of school-based CSE.

## Methods

The protocol of this scoping review was registered at the Open Science Framework (Registration DOI: 10.17605/OSF.IO/7CYZK). This scoping review is reported according to Preferred Reporting Items for Systematic reviews and Meta-Analyses extension for Scoping Reviews (PRISMA-ScR) [27]. The PRISMA-ScR checklist can be accessed in Supplementary Table S1.

### Criteria for considering studies for the review

#### Setting

This review included studies conducted in public or private schools. Other types of schools (e.g. alternative, vocational) were not included in this review due to their differing nature, objectives, curricula and teaching methods in comparison to public or private schools. Moreover, interventions implemented in a college or university setting were excluded from this review because the participants within those studies were older than the target age group of this review.

#### Types of studies

This review included randomized controlled trials (RCTs), cluster RCTs, quasi-RCTs, interrupted time series (ITS) and controlled before and after studies. There was no limitation of geographical area. We included studies published in the English language only.

#### Types of participants

The participants in this review were adolescents between the ages of 10 and 19 years defined by United Nations [28]. The majority of participants in this review were 19 years or younger but students older than 19 years from the same classes were also included because school-based interventions were conducted in classes of the same grade rather than targeted to a specific age group.

#### Types of interventions

This review focused on school-based CSE that aimed to prevent adolescent pregnancy. The educational interventions that were implemented within a context of a multisession curriculum, which took place in schools were included. Interventions that provided information on at least one of the following topics: pregnancy, abortion, contraceptives (available methods, effectiveness, and appropriate method use), and HIV/STI prevention (including condom use) were included. The intervention sessions were either didactic or participatory, with or without the use of technology and without any restrictions on the provider of the intervention. Additionally, the intervention may have been supplemented with parenting, services, or communication, and may have been presented in either print or digital format, or via interpersonal communication. The comparison group either received routine sexuality education or no intervention.

#### Types of outcome measures

##### Primary outcomes

Studies that reported one of the following outcomes were included.

- Contraceptive use during the last sex
- Unintended Pregnancy
- Abortion

In order to measure the impact of interventions on contraceptive use, a minimum follow up of three months after the intervention was required. For pregnancy and abortion, the minimum time required to measure these outcomes was six months after the intervention. However, if the prespecified follow up was unavailable, the follow up period defined by the authors was utilized. In case where contraceptive use was assessed in various ways, the focus was on the contraceptive use during the last sex. If none of the prespecified outcomes were reported, the outcome(s) defined by the authors were included, e.g. sex without effective pregnancy prevention.

##### Secondary outcomes

- Knowledge of contraception or contraceptive effectiveness
- Awareness of contraceptive methods
- Attitude toward contraception or a specific contraceptive method

The time frame for evaluating these secondary outcomes was determined by the definitions of the outcomes, as defined by the authors.

### Search methods for identification of studies

#### Electronic searches

We performed a systematic literature search from the existence of each database to 4th Nov 2023 using major electronic databases, including 1) PubMed, 2) the Cochrane Central Register of Controlled Trials (CENTRAL), 3) Scopus, 4) ISI Web of Science, and 5) Cumulative Index to Nursing and Allied Health Literature (CINAHL), to identify potential eligible studies. We also searched for potential eligible ongoing studies in the clinicaltrials.gov and WHO International Clinical Trials Registry Platform (WHO ICTRP). Full search strategies are presented in Supplementary Table S2.

#### Searching other sources

We checked the reference lists of relevant systematic reviews to identify potential eligible studies. Internet searches for the websites and organizations (e.g. Guttmacher Institute, CDC, WHO, UNFPA, UNESCO, etc.) were also done to identify articles for evidence-based sexuality education programs. In addition, we conducted Google scholar searches and screened the first 50 results to identify sexuality education programs and policies led by government bodies or other agencies and organizations relevant to adolescent health.

#### Study selection

All the titles and abstracts of studies obtained from the electronic database searches were checked and deduplicated in Mendeley software. Following the screening of titles and abstracts, the full texts of studies that met the eligibility criteria were reviewed using the Rayyan software. All the processes were done independently by two review authors (SMM, JS, and KLS). Any discrepancies were discussed and if necessary, consultation of a third person was sought (PL, PP, or CN). PRISMA flow diagram was used to illustrate a summary of the study selection process.

#### Data collection

The data extraction form using Microsoft Excel was tested with ten randomly selected studies independently (SMM and JS) and checked for consistency. Two review authors (SMM and KLS) extracted data from the included studies independently into the data extraction form. We extracted the following information: participants’ characteristics (e.g. age, gender, etc.), countries and settings (e.g. types of school, etc.), interventions (e.g. concepts, duration, providers, etc.), outcomes of interest (e.g. contraceptive use, unintended pregnancy, etc.), and study designs (e.g. RCTs, quasi-RCTs, etc.). Any differences between reviewers were solved through discussion or by asking the opinion from the third reviewer (PL, PP, or CN) if necessary. If additional information or clarification was required, we contacted the first and corresponding authors of the studies.

#### Synthesis of results

The extracted data were summarized using frequency and percentage for categorical variables. We presented the findings in figure, map and table. The findings were categorized and reported according to the following themes:

1. Study characteristics: country and setting, type of studies and participants
2. School-based CSE: components, mode of delivery, concepts, duration and provider
3. Outcomes reported in the included studies: primary outcomes included contraceptive use during the last sex, unintended pregnancy, abortion and secondary outcomes included knowledge of contraception or contraceptive effectiveness, awareness of contraceptive methods and attitude toward contraception or a specific contraceptive method

## Results

### Results of the search

**Fig. 1:**
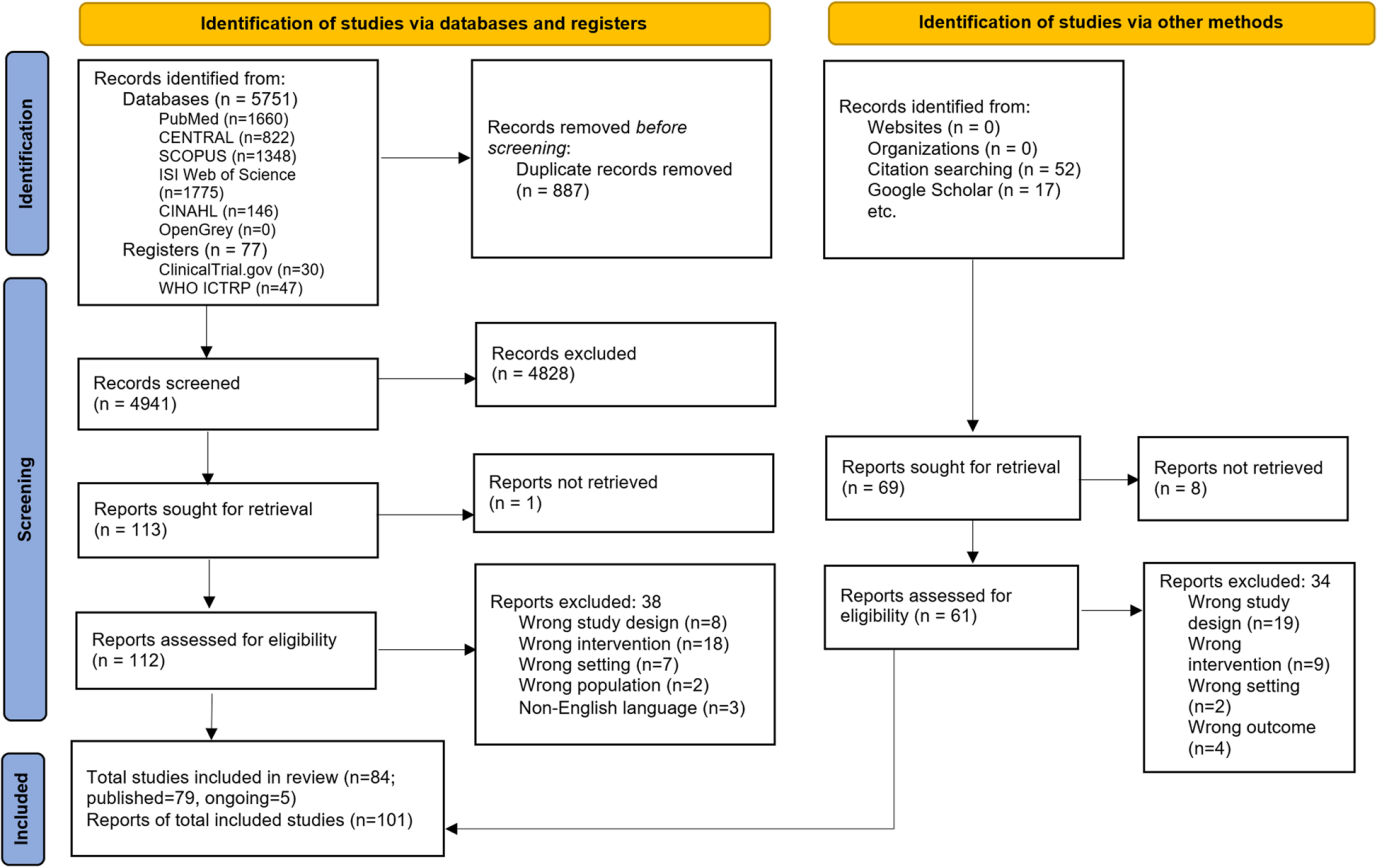
PRISMA flow chart

The screening process of the study is summarized in the PRISMA flow diagram (Fig. 1). The searches yielded 5828 records from major electronic databases and registers and 69 additional records from citation searches and Google scholar.

Among records identified from electronic databases, after removing 887 duplicates, a total of 4941 records were screened. We excluded 4828 records after screening the titles and abstracts. We reviewed 112 of 113 full texts since one full text could not be identified. Authors of three studies were contacted for more information and two provided publications of the studies. Thirty-eight reports were excluded due to ineligible study design (8 reports), ineligible intervention (18 reports), ineligible setting (7 reports), ineligible population (2 reports), and non-English language (3 reports).

We were only able to download the full texts for 61 of the reports identified from other sources. Of these, 34 reports did not meet the inclusion criteria of this review: due to ineligible study design (19 reports), ineligible interventions (9 reports), ineligible settings (2 reports), and ineligible outcomes (4 reports). Subsequently, 84 studies (101 reports) were found to be eligible for inclusion in this scoping review, five of these studies are ongoing studies (Supplementary Table S3). Therefore, a total of 79 studies were included in this review (Supplementary Table S4) and the characteristics of these included studies are described in Supplementary Table S5.

### Description of included studies

In this section, we have provided an overview of the key characteristics of the included studies, based on countries and settings as well as type of studies, participants, interventions and outcomes (Supplementary Table S5).

#### Countries and settings

The majority of the included studies were conducted in the United States (30 studies, 40%), followed by South Africa (7 studies), United Kingdom (5 studies), Nigeria (4 studies), Tanzania (3 studies), Spain (3 studies), Zambia (3 studies) and Bahamas, Ethiopia, Indonesia, Ghana, Mexico, Uganda (2 studies each) (Supplementary Figure S1). According to the 2022 World Bank classification [29], 43 studies (54.4%) were from high income countries (HICs), 13 from upper-middle income countries (UMICs), 16 from lower-middle income countries (LMICs), and seven from low income countries (LICs). Twenty-seven studies (3.2%) were conducted in high schools, another 27 studies (3.2%) in secondary/middle schools and seven studies (8.9%) in elementary/primary schools. However, 18 studies (22.8%) did not specify the type of schools. The number of schools involved in the included studies ranged from one to 157 schools.

#### Type of studies

The majority of the included studies were cluster RCTs (57 studies, 72.2%) followed by 20 quasi-RCTs (25.3%), and two RCTs (2.5%). ITS and before and after study could not be identified. The studies were published between 1986 and 2023. Thirty studies (38.0%) were published before 2010, 13 studies (16.3–5%) between 2010 and 2015, and 36 studies (45.6%) after 2015.

#### Participants

Participants in most included studies (77 studies, 97.5%) were adolescents between the age of 10 and 19 years. However, two studies also included older participants (10–24 years and 15–30 years) because although interventions were implemented for adolescents, they were followed up until 24 or 30 years of age. Sixty-eight studies included both genders while eight studies were exclusively targeted towards girls, and one study focused on boys only. Sample sizes across studies ranged from 125 to over 15,000 students.

#### Interventions

A wide variety of interventions were evaluated among the included studies. Twenty-eight studies (35.4%) were aimed at delaying sexual initiation, promoting safe sex behavior, preventing pregnancy, while 37 studies (46.8%) aimed at preventing HIV/STI and 14 studies (17.7%) targeted the prevention of both pregnancy and HIV. One intervention focused on emergency contraception, and some interventions were designed to be combined with other topics, such as drug abuse in three studies and alcohol in two studies. Among included studies which reported primary outcomes, only a few revealed culturally sensitive interventions indirectly but none of them had ever mentioned any aspect of religion. Twenty-nine studies used standard or existing sex education and 16 studies used health promotion as the control. The interventions with more than two arms were observed in 15 studies (18.9%).

All studies included participatory activities, such as discussion, group work and self-directed learning (e.g. demonstration and role play). Sixteen studies (20.3%) used films or videos, two studies used webisodes or online content, two used computer-based intervention, two used media and three used magazine or book for teaching aids. In sixteen studies (20.3%), the multi-component interventions involving education, school committee, community and services were reported. Interventions for parents were included in eleven studies (13.9%). Health services for students were integrated in eight studies (10.1%), while three interventions offered counseling. The community component was integrated in five studies and three studies used students’ clubs.

**Fig. 2:**
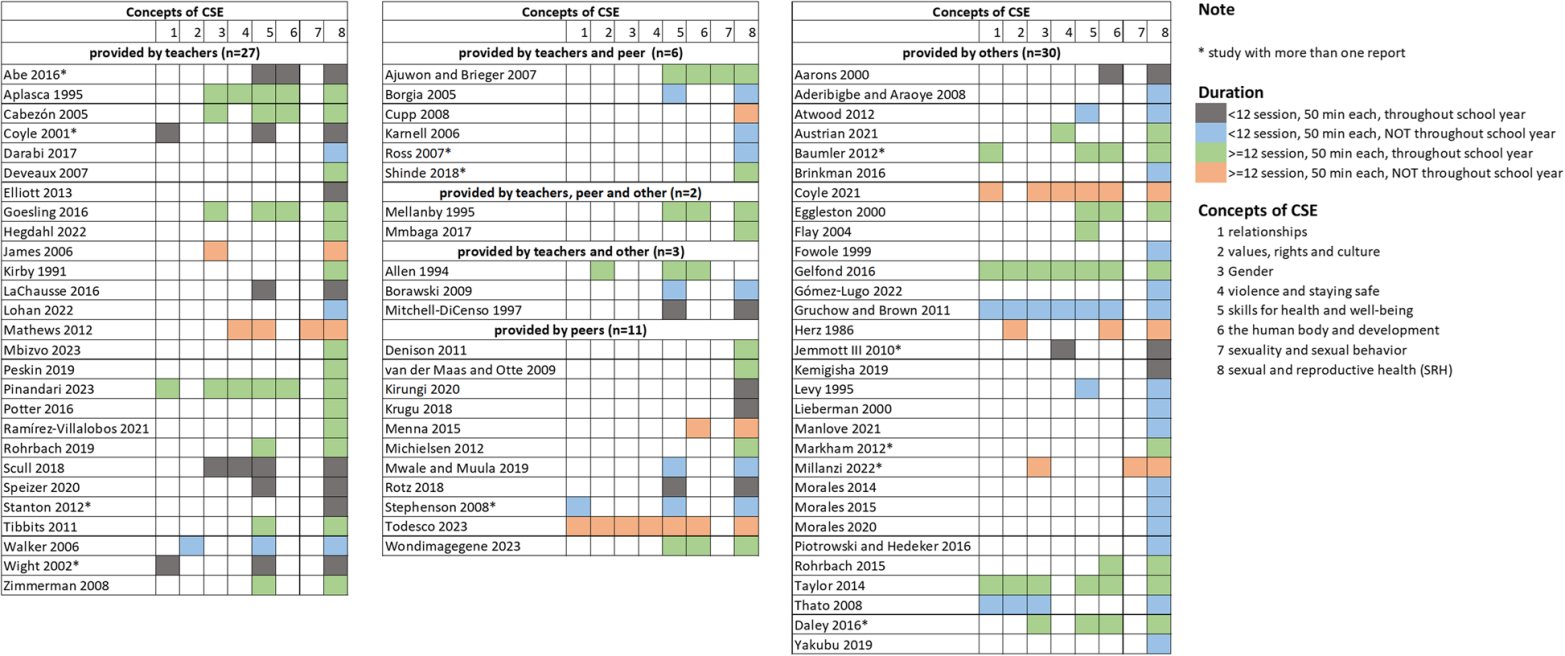
Concepts of School-based Comprehensive Sexuality Education (CSE) categorized by the intervention providers

Figure 2 provides the CSE concepts categorized by the intervention providers. The first group comprises interventions delivered solely by teachers (27 studies, 34.1%), indicating the CSE concepts and the duration of the interventions. Other groups included interventions delivered by teachers in combination with peer or other facilitators, or delivered solely by peers. The last group (30 studies, 38.0%) includes interventions delivered by others such as facilitators, researchers, health educator, school health nurses, etc. Among the eight concepts of CSE, sexual and reproductive health (SRH) was included in almost all studies (77 studies, 97.5%), followed by skills for health and well-being (36 studies, 48.0%), the human body and development (21 studies, 28.0%), gender (14 studies, 18.7%), relationship (11 studies, 14.7%), values, rights and culture (eight studies, 10.7%), violence and staying safe (nine studies, 12.0%) and sexuality and sexual behavior (three studies, 4.0%) (Fig. 2).

**Fig. 3:**
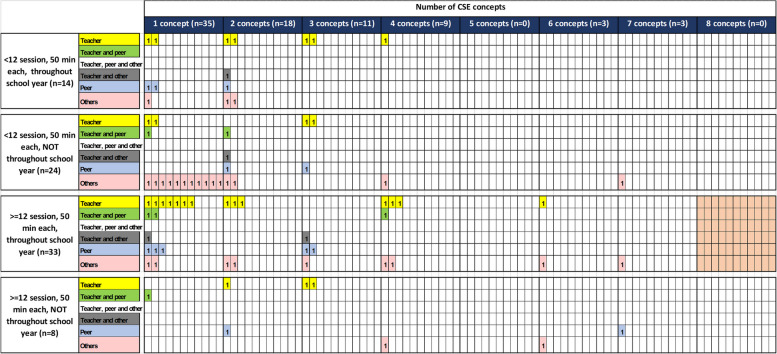
Summary of School-based Comprehensive Sexuality Education (CSE). Each square represents the number of included studies with duration of the intervention (rows) against number of CSE concepts (columns); additionally, the provider of the intervention is provided (color). The optional intervention is highlighted by the light orange rectangular on the right side

The evidence map presented in Fig. 3 provides a summary of school-based CSE, covering the concepts, duration (number of sessions, time of each session and the distribution of sessions) and providers of the interventions. Regarding the number of concepts covered, 35 studies (44.3%) of the intervention covered only one concept, while 18 studies (22.8%) covered two concepts, eleven studies (13.9%) covered three concepts, nine studies (11.4%) covered four concepts, and three studies, (3.7%) each covered for six and seven concepts (Fig. 3).

The interventions in the included studies varied in terms of session and duration of each session and distribution of these sessions throughout the school year and are provided in Fig. 3. Thirty-three studies (41.8%) had interventions with 12 or more sessions, each lasting for 50 min and were conducted throughout the school year which is recommended by ITGSE. Of these 33 studies, 15 studies provided only one CSE concept. Twenty-four studies (30.4%) had interventions with less than 12 sessions, each lasting 50 min, conducted only in some period of the school year (Fig. 3).

An optimal approach for delivering the eight concepts of CSE would involve teachers and other providers throughout the school year, with at least 12 sessions of 50 min each, to achieve the desired outcomes. This recommended approach is highlighted by the light orange rectangular on the right side of the Fig. 3. However, none of the interventions identified in this scoping review adhered to this recommended approach (Fig. 3).

#### Outcomes

Seventy-seven out of 79 included studies provided our prespecified primary and secondary outcomes and other outcomes. The summary of primary and secondary outcomes reported in the included studies by study design was presented in Table 1 and other outcomes by study design in Table 2.

**Table 1:** Included studies with prespecified primary and secondary outcomes by study designs

Outcomes	Number of included studies	RCT	Cluster RCTs	Quasi-RCTs
**Primary outcomes**				
Contraceptive use during the last sex	23	0	17	6
Unintended pregnancy	3	0	3	0
Abortion	3	0	3	0
**Secondary outcome**				
Knowledge of contraception or contraceptive effectiveness	25	1	20	4
Awareness of contraceptive methods	1	0	1	0
Attitude toward contraception or a specific contraceptive method	9	0	2	7

**Table 2:** Included studies with other outcomes by study designs

Outcomes	Number of studies	RCT	Cluster RCTs	Quasi-RCTs
**Contraceptive uptake**				
Contraceptive use	30	1	21	8
Contraceptives use at first sex	6	0	3	3
Contraceptive use in past three months	17	0	15	2
Condom use at last six months or more	4	0	2	2
**Pregnancy**				
Pregnancy	9	0	7	2
Unwanted pregnancy	1	0	1	0
Conception	1	0	1	0
Pregnant or made someone pregnant	3	0	2	1

### Primary outcomes

Twenty-three studies (17 cluster-RCTs and six quasi-RCTs) evaluated contraceptive use during the last sex. Seventeen studies implemented CSE alone, among these, four studies found that CSE was effective in increasing contraceptive use during the last sex. Among six studies which implemented CSE combined with other interventions (parent or community or services), three studies found that CSE was effective in increasing contraceptive use during the last sex. Three cluster RCTs reported unintended pregnancy. All showed a reduction in unintended pregnancy, two studies implemented CSE alone and the remaining one had CSE combined with services. Three cluster RCTs examined abortion. Two studies implemented CSE alone, and another one study combined CSE with access to services. None of these cluster RCTs showed significant impact of CSE on abortion (Table 1).

### Secondary outcomes

Knowledge of contraception or contraceptive effectiveness was reported in 25 studies including one RCT, 20 cluster RCTs and four quasi-RCTs, awareness of contraceptive methods was reported in one cluster RCT and attitude towards contraception or a specific contraceptive method was reported in nine studies (two cluster RCTs and seven quasi-RCTs) (Table 1).

### Other outcomes

The other non-prespecified outcomes reported in the included studies were contraceptive uptake (four items) and pregnancy (four items). Please see details in Table 2.

## Discussion

This scoping review presents a summary of the available evidence regarding school-based CSE and its effect on contraceptive use, unintended pregnancy and abortion among adolescents for prevention of adolescent pregnancy. This review shows gaps in school-based CSE implementation in terms of completeness of concepts, components, providers, duration and outcomes recommended by ITGSE.

CSE is a key for sustainable development, crucial in improving health, a pillar in delivering education of good quality and contributes to gender equality [30]. However, more than half of the studies included in this review were conducted in High-Income Countries (HICs), with two-fifth of the studies being conducted in the United States. CSE studies were concentrated in the US because more resources are available for conducting research, including funding, experienced researchers and access to advanced technologies and research infrastructure. Moreover, the US has a policy focusing on adolescent pregnancy prevention and a large amount of funding are available for sexuality education research, a culture that places a high value on scientific research and evidence-based policy making. Researchers seek to understand how different populations are affected by different approaches to sexuality education because US is a large country with diverse communities and cultures [31].

About three-quarters of the included studies used cluster RCTs as their study design which were appropriate because of the feasibility, practicality, and avoidance of risk of contamination between intervention and control groups. However, the authors should use appropriate statistical methods at the sample size calculation and analysis to take account for clustering effect.

All included studies were school-based and focused on all adolescent students in general and no special consideration was given to their diverse sexual and gender identities.

Sexuality education would be more impactful when school-based program is complemented with adolescent friendly health services, parental engagement, and community involvement [6, 32, 33]. However, in this scoping review, only one fifth of studies used multicomponent interventions. This might be because of scarcity of resources to be multicomponent. Although school is the best platform for CSE intervention, it should be integrated with community and health services involvement. This is worth to be seriously considered in the future CSE studies.

None of the included studies covered all eight concepts of CSE per the ITGSE. Nearly half of the included studies covered only one concept. Almost all studies covered Sexual and Reproductive Health which is the eighth concept of CSE. The most common concept was “Sexual and Reproductive Health” followed by “Skill for Health and Well-being” and the least common was “Sexuality and Sexual Behavior”. Each of the eighth concept of CSE covers two to five topics, each topic has key ideas and learning objectives to improve knowledge, attitude, and skills [6]. However, we could not get detail information about the topics and the key ideas for each concept stated in ITGSE. In addition, the modification and adaptation of CSE with regards to religious and cultural context is key for effective CSE but many had failed to indicate these social contexts in the intervention.

The delivery of sexuality education is very important to be able to develop the skills of adolescents needed to support healthy choices. Planning and implementing CSE is complex [33]. The participatory teaching methods and self-directed learning ensures adolescents to actively involve and help them internalize and integrate information [6, 32]. Almost all interventions in this review described participatory activities. The role of digital media as a delivery mechanism should be considered in the age of technology and the innovative, creative approaches by adolescents for adolescents will magnify the effect of sexuality education. Only some studies in this review incorporated webisodes or online, computer and media.

The teachers were the providers for CSE in one third of the included studies. However, for CSE, capable and motivated educators are essential [6]and there should be more investment in teacher education and support [33]. We need to consider the teachers training (preservice and in service) with accessible resources and support to deliver the best quality sexuality education in schools.

The included studies reported a heterogeneous range of outcomes which hinder reliable comparison between studies. Besides, very few studies reported the prespecified primary outcomes of contraceptive use during last sex, unintended pregnancy, and abortion and hence this highlighted the gaps of available evidence for these outcomes. Therefore, the standard outcomes are needed for measuring and comparing the CSE interventions. Two-fifths of the studies who reported primary outcomes were effective and among them, two-fifths of the studies implemented multicomponent CSE.

In conclusion, duration, concepts, teaching methods and providers varied across studies. Though, some degree of school-based CSE effectiveness on contraceptive use during the last sex and unintended pregnancy was reported, none of the included study implemented the recommended CSE approach showing the prominent gaps of the school-based CSE implementation.

### Strengths and limitations

To the best of our knowledge, this is the first scoping review to identify and map the available evidence of school based CSE for prevention of adolescent pregnancy with ITGSE globally. The review included all interventions related to promoting safe sex behavior, preventing pregnancy and HIV/STI and was not restricted by publication year. The screening and data extraction were done independently by two review authors. The review team met regularly and discussed how to resolve queries and concerns and ensured every step in a systematic way. However, there are some limitations. Due to limited time and resources, only publications in English were included. Furthermore, only the references list of systematic reviews but not all included studies were searched.

### Implications for future practice and research

We recommend a further systematic review to evaluate the effectiveness of school-based CSE. However, the heterogeneity of outcome measures across studies might be a challenge to performing a meta-analysis. To address this, it is important to establish standardized key outcome set, also known as CORE outcome set, for evaluating the effectiveness of school-based CSE interventions.

Our review highlights the need for well-defined school-based multicomponent programs that cover all ITGSE concepts and have an appropriate duration provided by teachers, particularly in LMICs. The study design should be cluster rather than individual RCTs using CORE outcome set.

## Conclusion

This scoping review provides the overview of the currently available evidence on school-based CSE aimed to prevent adolescent pregnancy. This review shows gaps in school-based CSE implementation in terms of completeness of concepts, components, providers, duration and outcomes recommended by ITGSE.

### Supplementary Information

**Supplementary material 1.**

## Data Availability

All data generated or analyzed during this study are included in this published article or the supplementary files.

## Abbreviations

CDC: Centers for Disease Control and Prevention
CENTRAL: Cochrane Central Register of Controlled Trials
CINAHL: Cumulative Index to Nursing and Allied Health Literature
Cluster RCTs: Cluster Randomized Controlled Trials
CSE: Comprehensive Sexuality Education
HICs: High Income Countries
HIV: Human Immunodeficiency Virus
ITGSE: International Technical Guidance on Sexuality Education
ITS: Interrupted Time Series
LICs: Low Income Countries
LMICs: Low- and Middle-Income Countries
MMC: Medical Male Circumcision
PRISMA: Preferred Reporting Items for Systematic Reviews and Meta-Analyses
PRISMA-ScR: Preferred Reporting Items for Systematic Reviews and Meta-Analyses Extension for Scoping Reviews
Quasi-RCTs: Quasi-Randomized Controlled Trials
RCTs: Randomized Controlled Trials
SDG: Sustainable Development Goals
SRH: Sexual and Reproductive Health
SRHR: Sexual and Reproductive Health and Rights
STI: Sexually Transmitted Infections
UMICs: Upper-Middle Income Countries
UNESCO: United Nations Educational, Scientific and Cultural Organization
UNFPA: United Nations Population Fund
US: United States
VIP: Virtual Infant Parenting
WHO: World Health Organization
WHO ICTRP: WHO International Clinical Trials Registry Platform
