# Supplementary material for: School-based comprehensive sexuality education for prevention of adolescent pregnancy: a scoping review
Source: BMC Womens Health. 2024 Feb 21;24:137. doi: 10.1186/s12905-024-02963-x (PMC10882910; doi:10.1186/s12905-024-02963-x)
Supplement: Supplementary file 1 — Supplementary material 1. [file 12905_2024_2963_MOESM1_ESM.docx]

# Title: School-based Comprehensive Sexuality Education for Prevention of Adolescent Pregnancy: A Scoping Review

## Authors:

Su Mon Myat^1,2^, Porjai Pattanittum^3^, Jen Sothornwit ^4^, Chetta Ngamjarus^3^, Siwanon Rattanakanokchai^3^, Kyaw Lwin Show^5,2^, Nampet Jampathong^6^, Pisake Lumbiganon^4^

## Affiliations:

^1^School Health Division, Department of Public Health, Ministry of Health, Myanmar

^2^Doctor of Epidemiology and Biostatistics Program, Department of Epidemiology and Biostatistics, Faculty of Public Health, Khon Kaen University, Thailand

^3^Department of Epidemiology and Biostatistics, Faculty of Public Health, Khon Kaen University, Thailand

^4^Department of Obstetrics and Gynecology, Faculty of Medicine, Khon Kaen University, Thailand

^5^Department of Medical Research, Ministry of Health, Myanmar

^6^Cochrane Thailand, Khon Kaen University, Thailand

### *Corresponding author:

**Name:** Dr. Porjai Pattanittum,

Address: Associate Professor, Department of Epidemiology and Biostatistics, Faculty of Public Health, Khon Kaen University, Thailand

Telephone: +66 862300217

Email: [pporja@kku.ac.th](mailto:pporja@kku.ac.th)

**Supplementary Information**

**Additional file 1:**

**Supplementary Figure S1. Distribution of countries of included studies**

**Supplementary Table S1. PRISMA-ScR checklist.**

**Supplementary Table S2. Search strategies.**

**Supplementary Table S3. Characteristics of ongoing studies.**

**Supplementary Table S4. List of included studies.**

**Supplementary Table S5. Characteristics of included studies.**


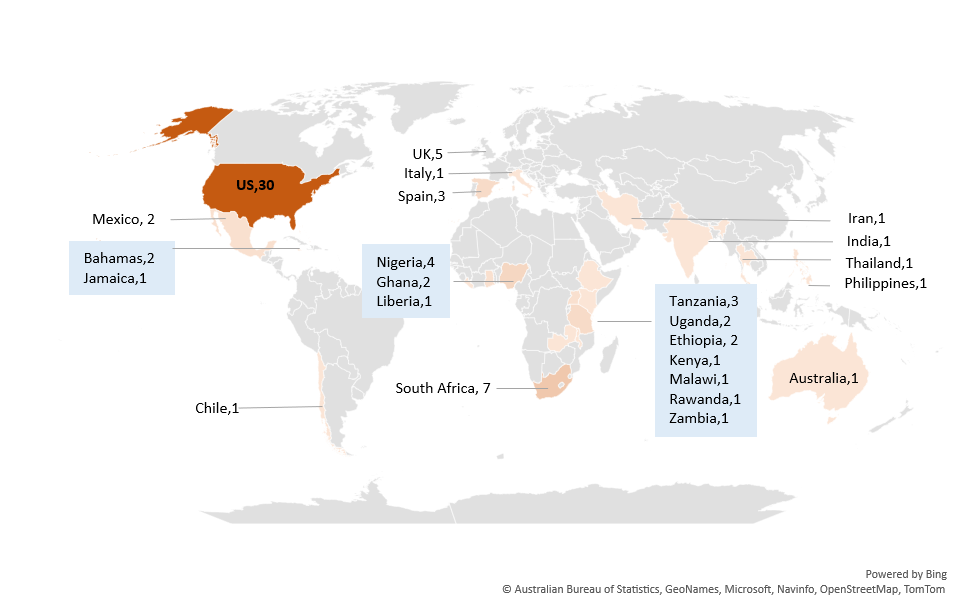


**Supplementary Figure S1. Distribution of countries of included studies**

**Supplementary Table S1:** PRISMA-ScR checklist

| **Section** | **Item** | **PRISMA-ScR Checklist Item** |  |
| --- | --- | --- | --- |
| **Title** | | | |
| Title | 1 | Identify the report as a scoping review | Title |
| **Abstract** | | | |
| Structured summary | 2 | Provide a structured summary that includes (as applicable): background, objectives, eligibility criteria, sources of evidence, charting methods, results, and conclusions that relate to the review questions and objectives | Abstract |
| **Introduction** | | | |
| Rationale | 3 | Describe the rationale for the review in the context of what is already known. Explain why the review questions/objectives lend themselves to a scoping review approach. | Background Rationale for undertaking a scoping review |
| Objectives | 4 | Provide an explicit statement of the questions and objectives being addressed with reference to their key elements (e.g., population or participants, concepts, and context) or other relevant key elements used to conceptualize the review questions and/or objectives. | Objectives |
| **Methods** | | | |
| Protocol and registration | 5 | Indicate whether a review protocol exists; state if and where it can be accessed (e.g., a Web address); and if available, provide registration information, including the registration number. | Methods |
| Eligibility criteria | 6 | Specify characteristics of the sources of evidence used as eligibility criteria (e.g., years considered, language, and publication status), and provide a rationale. | Methods |
| Information sources | 7 | Describe all information sources in the search (e.g., databases with dates of coverage and contact with authors to identify additional sources), as well as the date the most recent search was executed. | Methods and Supplementary Table S2 |
| Search | 8 | Present the full electronic search strategy for at least 1 database, including any limits used, such that it could be repeated | Supplementary Table S2 |
| Selection of  sources of  evidence | 9 | State the process for selecting sources of evidence (i.e., screening and eligibility) included in the scoping review. | Methods |
| Data charting  process | 10 | Describe the methods of charting data from the included sources of evidence (e.g., calibrated forms or forms that have been tested by the team before their use, and whether data charting was done independently or in duplicate) and any processes for obtaining and confirming data from investigators. | Methods |
| Data items | 11 | List and define all variables for which data were sought and any assumptions and simplifications made. | Methods |
| Synthesis of results | 13 | Describe the methods of handling and summarizing the data that were charted | Methods |
| **Results** | | | |
| Selection of sources of evidence | 14 | Give numbers of sources of evidence screened, assessed for eligibility, and included in the review, with reasons for exclusions at each stage, ideally using a flow diagram. | Methods |
| Characteristics of sources of  evidence | 15 | For each source of evidence, present characteristics for which data were charted and provide the citations. | Methods and Supplementary Table S2, S3 |
| Results of individual sources of evidence | 17 | For each included source of evidence, present the relevant data that were charted that relate to the review questions and objectives | Not reported |
| Synthesis of results | 18 | Summarize and/or present the charting results as they relate to the review questions and objectives | Results, Figure 1,2,3,4 and Table 1 |
| **Discussion** | | | |
| Summary of evidence | 19 | Summarize the main results (including an overview of concepts, themes, and types of evidence available), link to the review questions and objectives, and consider the relevance to key groups | Discussion |
| Limitations | 20 | Discuss the limitations of the scoping review process. | Strengths and Limitations |
| Conclusions | 21 | Provide a general interpretation of the results with respect to the review questions and objectives, as well as potential implications and/or next steps | Conclusion |
| **Funding** | | | |
| Funding | 22 | Describe sources of funding for the included sources of evidence, as well as sources of funding for the scoping review. Describe the role of the funders of the scoping review | sources of funding for the included sources of evidence not described, sources of funding for the scoping review reported |

**Supplementary Table S2: Search strategies**

Database: **PudMed**

Date search conducted: 4 Nov 2023

| **Search** | **Query** |
| --- | --- |
| #1 | adolescent [MeSH Terms] |
| #2 | child [MeSH Terms] |
| #3 | young adult [MeSH Terms] |
| #4 | adolescent* OR "adolescence" OR teen* OR teenager* OR youth* OR juvenile* OR child* OR "boy" OR "boys" OR girl* OR schoolboy* OR schoolgirl* OR "young man" OR "young men" OR "young lady" OR "young ladies" OR youngster* |
| #5 | #1 OR #2 OR #3 OR #4 |
| #6 | sexuality education [MeSH Terms] AND (school health service [MeSH Terms] OR school [MeSH Terms]) |
| #7 | "comprehensive sexuality education" OR "CSE" |
| #8 | "sex" OR sexual* OR "family planning" OR reproduct* |
| #9 | educat* OR "training" OR instructor* OR counsel* OR teach* OR coach* OR mentor* OR lectur* OR tutor* OR learn* |
| #10 | school* OR "classroom" OR "class room*" |
| #11 | #8 AND #9 |
| #12 | (#7 OR #11) AND #10 |
| #13 | #6 OR #12 |
| #14 | contraception [MeSH Terms] |
| #15 | (("fertilization" OR "fertility" OR "birth") AND ("inhibition" OR control* OR block* OR prevent*)) OR "contraceptive method*" OR contraception* OR "family planning" OR "contraceptive agent*" OR "contraceptive device*" OR "contraceptive drug*" OR "contraceptive pill*" OR "condom" |
| #16 | pregnancy [MeSH Terms]OR pregnancy in adolescence [MeSH Terms] |
| #17 | pregnanc* OR gestation* |
| #18 | abortion* |
| #19 | unintended pregnancy[MeSH Terms] |
| #20 | “unintended pregnancy” |
| #21 | #14 OR #15 OR #16 OR #17 OR #18 OR #19 OR #20 |
| #22 | randomized controlled trial [PT] OR controlled before-after studies [MeSH Terms] |
| #23 | ((("randomized" OR "randomised") AND "controlled trial*") OR (("randomized" OR "randomised") AND "control trial*") OR "RCT" OR "randomly") OR ("before and after" OR CBA OR "before-after") |
| #24 | Interrupted time series [MH] |
| #25 | "time series" OR ("interrupted" AND "time series") OR ("ITS" AND ("studies" OR "study")) |
| #26 | quasi randomized control trial[MeSH Terms] |
| #27 | “quasi randomized control trial” |
| #28 | “Cluster randomised controlled trial*” OR “Cluster randomised trial*” OR “group-randomised trial*” |
| #29 | #22 OR #23 OR #24 OR #25 OR #26 OR #27 OR #28 |
| #30 | #5 AND #13 AND #21 AND #29 |

Database: **Cochrane Library**

Date search conducted: 4 Nov 2023

| **Search** | **Query** |
| --- | --- |
| #1 | MeSH descriptor: [Adolescent] explode all trees |
| #2 | MeSH descriptor: [Child] explode all trees |
| #3 | MeSH descriptor: [Young Adult] explode all trees |
| #4 | adolescent* OR "adolescence" OR teen* OR teenager* OR youth* OR juvenile* OR child* OR "boy" OR "boys" OR girl* OR schoolboy* OR schoolgirl* OR "young man" OR "young men" OR "young lady" OR "young ladies" OR youngster* |
| #5 | #1 OR #2 OR #3 OR #4 |
| #6 | MeSH descriptor: [Sexuality education] explode all trees |
| #7 | "comprehensive sexuality education" OR "CSE" |
| #8 | "sex" OR sexual* OR "family planning" OR reproduct* |
| #9 | educat* OR "training" OR instructor* OR counsel* OR teach* OR coach* OR mentor* OR lectur* OR tutor* OR learn* |
| #10 | school* OR "classroom" OR (class room*) |
| #11 | MeSH descriptor: [School Health Services] explode all trees |
| #12 | (#8 AND #9) OR #6 OR #7 |
| #13 | (#10 OR #11) AND #12 |
| #14 | MeSH descriptor: [Contraception] explode all trees |
| #15 | MeSH descriptor: [Pregnancy] explode all trees |
| #16 | MeSH descriptor: [Pregnancy in Adolescence] explode all trees |
| #17 | pregnanc* OR gestation* |
| #18 | (("fertilization" OR "fertility" OR "birth") AND ("inhibition" OR control* OR block* OR prevent*)) OR (contraceptive method*) OR contraception* OR "family planning" OR (contraceptive agent*) OR (contraceptive device*) OR (contraceptive drug*) OR (contraceptive pill*) OR "condom" |
| #19 | “Unintended pregnancy” |
| #20 | MeSH descriptor: [Pregnancy, Unplanned] explode all trees |
| #21 | #14 OR #15 OR #16 OR #17 OR #18 OR #19 OR #20 |
| #22 | #5 AND #13 AND #21 AND #28 in Trials |

Database: **Scopus**

Date search conducted: 4 Nov 2023

| **Search** | **Query** |
| --- | --- |
| #1 | TITLE-ABS-KEY(adolescent* OR "adolescence" OR teen* OR teenager* OR youth* OR juvenile* OR child* OR "boy" OR "boys" OR girl* OR schoolboy* OR schoolgirl* OR "young man" OR ("young men") OR ("young lady") OR ("young ladies") OR youngster* OR (“young adult”)) |
| #2 | TITLE-ABS-KEY(school* OR "classroom" OR "class room*") |
| #3 | TITLE-ABS-KEY("sex" OR sexual* OR ("family planning") OR reproduct*) |
| #4 | TITLE-ABS-KEY(educat* OR “training” OR instructor* OR counsel* OR teach* OR coach* OR mentor* OR lectur* OR tutor* OR learn*) |
| #5 | TITLE-ABS-KEY(("comprehensive sexuality education") OR "CSE") |
| #6 | (#3 AND #4) OR #5 |
| #7 | #2 AND #6 |
| #8 | TITLE-ABS-KEY((("fertilization" OR "fertility" OR "birth") AND ("inhibition" OR control* OR block* OR prevent*)) OR ("contraceptive method*") OR contraception* OR ("family planning") OR ("contraceptive agent*") OR ("contraceptive device*") OR ("contraceptive drug*") OR ("contraceptive pill*") OR "condom" OR pregnanc* OR gestation* OR abortion* OR (“unintended pregnancy”)) |
| #9 | TITLE-ABS-KEY (( "randomized controlled trial" OR ( "Controlled before-after studies" ) OR ( "Interrupted time series" ) OR ( "ITS" ) OR ( "time series" ) OR ( “quasi AND randomized AND control AND trial” ) OR ( “cluster AND randomised AND controlled AND trial*” ) OR ( “cluster AND randomised AND trial*” ) OR ( “group-randomised AND trial*” ) )) |
| #10 | #1 AND #7 AND #8 AND #9 |

Database: **ISI Web of Science**

Date search conducted: 12 May 2022

| **Search** | **Query** |
| --- | --- |
| #1 | TS=(adolescent* OR "adolescence" OR teen* OR teenager* OR youth* OR juvenile* OR child* OR "boy" OR "boys" OR girl* OR schoolboy* OR schoolgirl* OR "young man" OR "young men" OR "young lady" OR "young ladies" OR youngster* OR “young adult”) |
| #2 | TS=((("sex" OR sexual* OR "family planning" OR reproduct*) AND (educat* OR "training" OR instructor* OR counsel* OR teach* OR coach* OR mentor* OR lectur* OR tutor* OR learn*)) OR (("comprehensive sexuality education" OR "CSE") AND (school* OR "classroom" OR "class room*"))) |
| #3 | TS=((("fertilization" OR "fertility" OR "birth") AND ("inhibition" OR control* OR block* OR prevent*)) OR "contraceptive method*" OR contraception* OR "family planning" OR "contraceptive agent*" OR "contraceptive device*" OR "contraceptive drug*" OR "contraceptive pill*" OR "condom" OR pregnanc* OR gestation* OR abortion* OR “unintended pregnancy”) |
| #4 | TS=("randomized controlled trial" OR "Controlled before-after studies" OR "Interrupted time series" OR "ITS" OR "time series" OR “quasi randomized control trial” OR “Cluster randomised controlled trial*” OR “Cluster randomised trial*” OR “group-randomised trial*”) |
| #5 | #1 AND #2 AND #3 AND #4 |

Database: **CINAHL**

Date search conducted: 4 Nov 2023

| **Search** | **Query** |
| --- | --- |
| S1 | TI ( adolescent* OR "adolescence" OR teen* OR teenager* OR youth* OR juvenile* OR child* OR "boy" OR "boys" OR girl* OR schoolboy* OR schoolgirl* OR "young man" OR "young men" OR "young lady" OR "young ladies" OR youngster* OR “young adult” ) OR AB ( adolescent* OR "adolescence" OR teen* OR teenager* OR youth* OR juvenile* OR child* OR "boy" OR "boys" OR girl* OR schoolboy* OR schoolgirl* OR "young man" OR "young men" OR "young lady" OR "young ladies" OR youngster* OR “young adult” ) |
| S2 | TI ( ((("sex" OR sexual* OR "family planning" OR reproduct*) AND (educat* OR "training" OR instructor* OR counsel* OR teach* OR coach* OR mentor* OR lectur* OR tutor* OR learn*)) OR ("comprehensive sexuality education" OR "CSE")) AND (school* OR "classroom" OR "class room*") ) OR AB ( ((("sex" OR sexual* OR "family planning" OR reproduct*) AND (educat* OR "training" OR instructor* OR counsel* OR teach* OR coach* OR mentor* OR lectur* OR tutor* OR learn*)) OR ("comprehensive sexuality education" OR "CSE")) AND (school* OR "classroom" OR "class room*") ) |
| S3 | TI ( (("fertilization" OR "fertility" OR "birth") AND ("inhibition" OR control* OR block* OR prevent*)) OR "contraceptive method*" OR contraception* OR "family planning" OR "contraceptive agent*" OR "contraceptive device*" OR "contraceptive drug*" OR "contraceptive pill*" OR "condom" OR pregnanc* OR gestation* OR abortion* OR “unintended pregnancy” ) OR AB ( (("fertilization" OR "fertility" OR "birth") AND ("inhibition" OR control* OR block* OR prevent*)) OR "contraceptive method*" OR contraception* OR "family planning" OR "contraceptive agent*" OR "contraceptive device*" OR "contraceptive drug*" OR "contraceptive pill*" OR "condom" OR pregnanc* OR gestation* OR abortion* OR “unintended pregnancy” ) |
| S4 | TI ( "randomized controlled trial" OR "Controlled before-after studies" OR "Interrupted time series" OR "ITS" OR "time series" OR “quasi randomized control trial” OR “Cluster randomised controlled trial*” OR “Cluster randomised trial*” OR “group-randomised trial*” ) OR AB ( "randomized controlled trial" OR "Controlled before-after studies" OR "Interrupted time series" OR "ITS" OR "time series" OR “quasi randomized control trial” OR “Cluster randomised controlled trial*” OR “Cluster randomised trial*” OR “group-randomised trial*” ) |
|  | S1 AND S2 AND S3 AND S4 |

Database: **Open Grey** [**http://www.opengrey.eu/**](http://www.opengrey.eu/)

Date search conducted: 4 Nov 2023

( ( adolescent$ OR "adolescence" OR teen$ OR teenager$ OR youth$ OR juvenile$ OR child$ OR "boy" OR "boys" OR girl$ OR schoolboy$ OR schoolgirl$ OR "young man" OR ( "young men" ) OR ( "young lady" ) OR ( "young ladies" ) OR youngster$ OR ( “young AND adult” ) ) ) AND ( ( ( school$ OR "classroom" OR "class room$" ) ) AND ( ( ( ( "sex" OR sexual$ OR ( "family planning" ) OR reproduct$ ) ) AND ( ( educat$ OR "training" OR instructor$ OR counsel$ OR teach$ OR coach$ OR mentor$ OR lectur$ OR tutor$ OR learn$ ) ) ) OR ( ( ( "comprehensive sexuality education" ) OR "CSE" ) ) ) ) AND ( ( ( ( "fertilization" OR "fertility" OR "birth" ) AND ( "inhibition" OR control$ OR block$ OR prevent$ ) ) OR ( "contraceptive method$" ) OR contraception$ OR ( "family planning" ) OR ( "contraceptive agent$" ) OR ( "contraceptive device$" ) OR ( "contraceptive drug$" ) OR ( "contraceptive pill$" ) OR "condom" OR pregnanc$ OR gestation$ OR abortion$ OR ( “unintended AND pregnancy” ) ) ) AND ( ( ( "randomized controlled trial" OR ( "Controlled before-after studies" ) OR ( "Interrupted time series" ) OR ( "ITS" ) OR ( "time series" ) OR ( “quasi AND randomized AND control AND trial” ) OR ( “cluster AND randomised AND controlled AND trial$” ) OR ( “cluster AND randomised AND trial$” ) OR ( “group-randomised AND trial$” ) ) ) )

Database: **ClinicalTrials**

Date search conducted: 4 Nov 2023

Database: **Trial of Registration (ICTRP)**

Date search conducted: 4 Nov 2023

**Supplementary Table S3: Characteristics of ongoing studies**

**Study 1**

| Study name | **Evaluation of the Yes You Can…Make Smart Choices! Curriculum** |
| --- | --- |
| Methods | Design: matched‐pair, cluster‐randomization design |
|  | Location: New Jersey |
|  | Time frame: 2022-2025 |
|  | Sample size estimation and outcome of focus: |
|  | empower young people to create healthy relationships and delay sexual activity by providing them with evidence‐informed, medically sound, trauma‐informed education on both abstinence and contraception and to also teach five adult preparation subjects 1) healthy relationships 2) adolescent development 3) financial literacy 4) healthy life skills,  and 5) strengthening parent‐child communication |
| Participants | General with N: 3500 participants |
|  | Inclusion criteria: 9th grade student at a curriculum (intervention) or control school at the time of enrollment |
| Interventions | 1) Intervention: (1) curriculum only, (2) curriculum + text‐messaging, (3) text‐messaging only |
|  | 2) Control: normal classroom instruction. |
| Outcomes | Primary: Delay sexual intercourse [Time Frame: Six/Twelve months after completion of the curriculum] |
|  | Secondary: Sexual behavior intent, Communication skills, Attitudes toward dating violence, Financial literacy [Time Frame: Six/Twelve months after completion of the curriculum] |
|  | Follow-up: Six/Twelve months after completion of the curriculum |
| Notes | Information from <https://clinicaltrials.gov/ct2/show/NCT05320666> , and last updated at April 2022. Recruitment Status: Not yet recruiting |

**Study 2**

| Study name | **Evaluation of Big Decisions in Three South Texas School Districts** |
| --- | --- |
| Methods | Design: cluster randomized controlled trial |
|  | Location: four high schools from three rural and semi-rural school districts in Texas |
|  | Time frame: September 1, 2016 to December 15 2019 |
| Participants | General with N: 3170 participants |
|  | Inclusion criteria: Must be in ninth grade and enrolled in a participating class at a participating high school |
|  | Exclusion criteria: Lack of consent or assent |
| Interventions | 1) Intervention: Big Decisions is a promising teen pregnancy prevention intervention that consists primarily of abstinence-plus sexual health education curriculum and also includes a parent component offered to all families. |
|  | 2) Control: Youth Voices is a benign comparison intervention that meets for two class sessions over three to five weeks. Classroom teachers deliver Youth Voices to 9th grade students. |
| Outcomes | Primary: Use of condom or effective method of birth control last time you had sex [ Time Frame: One year ] |
|  | Secondary: intention to use condom or birth control [ Time Frame: immediate post-test ], Frequency of communication with parents about abstinence and sex [ Time Frame: immediate post-test ] |
|  | Follow-up: at baseline, immediately post-intervention, and at 12-month follow-up |
| Notes | Information from <https://clinicaltrials.gov/ct2/show/NCT04508569> reportedly completed Aug 2020, Recruitment-completed, last update-11Aug2020 |

**Study 3**

| Study name | **Evaluation of the Making Proud Choices! Teen Pregnancy Prevention Program** |
| --- | --- |
|  | The MPC curriculum aims to provide adolescents with the information and tools they need to reduce their risk of sexually transmitted diseases (STDs), HIV, and pregnancy. The curriculum emphasizes abstinence as the safest choice for avoiding pregnancy and STDs, but also encourages youth to use condoms and other methods of birth control if they do have sex. The curriculum includes video clips, student role-playing activities, and group discussions. It also involves skill-building activities for correct condom use, refusal techniques, and safer-sex negotiation, and opportunities for students to practice these skills. |
| Methods | Design: cluster randomized controlled trial (RCT) |
|  | Location: four geographic areas across the U.S |
|  | Time frame: (2016‐17, 2017‐18 and 2018‐19) completed |
|  | Sample size estimation and outcome of focus: |
| Participants | General with N: 2810 participants |
|  | Inclusion criteria: Enrolled in a study school, Attending the targeted class (e.g. health class) for the first time |
|  | Exclusion criteria: None |
| Interventions | 1) Intervention: MPC implemented by health educators |
|  | 2) Control: business as usual |
| Outcomes | Primary:  Knowledge about HIV  Knowledge about pregnancy  Knowledge about condoms  Knowledge about other forms of contraception  Belief that sex may adversely affect future goals  Belief that condoms can be pleasurable  Attitudes about condoms  Condom self-efficacy  Condom negotiation  Refusal skills  Ever any sex  Any sex (in past 3 months)  Times having any sex (in past 3 months)  Count of vaginal sex partners (in the past 3 months)  Any sex without a condom (in past 3 months)  Times having any sex without a condom in past 3 months  Sex without birth control in past 3 months  Times having sex without birth control (in past 3 months)  Ever pregnant  Any sexually transmitted infection (STI)  [ Time Frame: Survey administered 6 months post program ] |
|  | Secondary: None |
|  | Follow-up: Survey administered 6 months post program |
| Notes | Information from ClinicalTrials.gov <https://clinicaltrials.gov/ct2/show/NCT04863326> and central, which has intervention information; reportedly completed 29 April 2021 |

**Study 4**

| Study name | **The Positive Choices trial of a social marketing intervention to promote sexual health and reduce health inequalities among English secondary school students** |
| --- | --- |
| Methods | Design: Superiority Phase III cluster parallel-group randomized controlled trial |
|  | Location: United Kingdom, England |
|  | Time frame: August 2021 to March 2025 |
|  | Sample size estimation and outcome of focus: 50 schools; minimum 6440, maximum 8500 students, Non-competent first sex assessed among trial participants having sex for the first time between baseline and follow-up (33 months) |
| Participants | Inclusion criteria:  1. Students aged 12-16 years  2. Deemed competent to consent by schools  3. Moving from year 8 into year 11 during the trial  4. In English secondary schools (including faith schools, free schools, academies and private schools) excluding pupil referral units, schools for those with special educational needs and disabilities, and schools with poor Ofsted (government school inspectorate) inspections |
|  | Exclusion criteria:  1. Students not deemed competent to consent by schools or students in pupil referral units  2. Schools for those with special educational needs and disabilities  3. Schools with poor Ofsted inspections |
| Interventions | 1) Intervention: Behavioural |
|  | school health promotion council comprising staff and students to plan, launch, coordinate and oversee delivery; a student needs survey of year-8 students which provides evidence to inform local tailoring; a classroom curriculum addressing social and emotional skills and relationships and sexual health knowledge and skills delivered by school staff; student-run social-marketing campaigns facilitated by trained teachers; parent information; and review of school and other local sexual and reproductive health services to inform improvements. |
| Outcomes | Primary: Non-competent first sex assessed among trial participants having sex for the first time between baseline and follow-up (33 months) |
|  | Secondary:   1. Non-competent last sex 2. Age at sexual debut 3. Non-use of contraception at first and last sex among those reporting heterosexual intercourse 4. Number of sexual partners 5. Dating and relationship violence victimization 6. Self-reported diagnoses of sexually transmitted infections 7. Pregnancy and unintended pregnancy among girls 8. Initiation of pregnancy among boys 9. Health-related quality of life |
|  | Follow-up: 33 months |
| Notes | Information from <https://www.isrctn.com/ISRCTN16723909?q=bonell&filters=&sort=&offset=1&totalResults=4&page=1&pageSize=10&searchType=basic-search> reportedly completed October 2021, Recruitment end date:01/03/2022 |

**Study 5**

| Study name | **SaFE: a sexual health and healthy relationships intervention for further education** |
| --- | --- |
| Methods | Design: Pilot 2-arm cluster randomized trial with an embedded process evaluation |
|  | Location: United Kingdom |
|  | Time frame: January 2020 to March 2023 |
|  | Sample size estimation and outcome of focus: six schools of FE settings; |
| Participants | General with N: 1,000 students |
|  | Inclusion criteria:  1. All state-funded FE settings including community colleges and 6th forms attached to secondary schools will be eligible to participate, including private and Welsh medium schools  2. All students aged 16 and older enrolled at participating FE settings |
|  | Exclusion criteria:  1. Schools/colleges for those with learning disabilities  2. Settings with extended existing onsite service provision (e.g. STI testing) |
| Interventions | SaFE intervention: 1) offering free onsite sexual health (e.g. condoms, STI tests and pregnancy tests) and relationship services; 2) publicising these services and; 3) training FE staff how to promote sexual health and recognize and respond to DRV and sexual harassment. |
|  | Condition: Sexual health |
| Outcomes | Primary: 1. Unprotected intercourse at last intercourse measured using validated questions from the SHARE questionnaires within the SaFE Student Survey, at baseline and 12-months post-baseline  2. Self-report experience of DRV victimisation in the last 12 months measured using sCADRI within the SaFE Student Survey, at baseline and 12-months post-baseline |
|  | Secondary:  STI and pregnancy prevention methods used at last intercourse  Use of emergency contraception at last intercourse  Sexual regret at last intercourse  Sexual harassment taking place at FE settings in the last 12 months  Non-volitional sex in the last 12 months  DRV perpetration in the last 12 months  Health related quality of life  Self-reported awareness of services |
| Notes | Information from ISCTRN, reportedly completed March 2020, Last edited Dec 2020 Recruitment status: Suspended  <https://www.isrctn.com/ISRCTN54793810?q=&filters=conditionCategory:Not%20Applicable&sort=&offset=11&totalResults=1221&page=1&pageSize=100&searchType=basic-search> |

**Supplementary Table S4: List of included studies**

| **Aarons 2000**  Aarons SJ, Jenkins RR, Raine TR, El-Khorazaty MN, Woodward KM, Williams RL, et al. Postponing sexual intercourse among urban junior high school students-a randomized controlled evaluation. J Adolesc Heal. 2000;27:236–47. |
| --- |
| **Abe, 2016***  Abe Y, Barker LT, Chan V, Eucogco J. Culturally responsive adolescent pregnancy and sexually transmitted infection prevention program for middle school students in Hawai’i. Am J Public Health. 2016;106 September: S110–6.  Manaseri H, Roberts KD, Barker LT, Tom T. Pono Choices: Lessons for School Leaders From the Evaluation of a Teen Pregnancy Prevention Program. J Sch Health. 2019;89:246–56. |
| **Aderibigbe 2008**  Aderibigbe SA, Araoye MO. Effect of health education on sexual behaviour of students of public secondary schools in Ilorin, Nigeria. Eur J Sci Res. 2008; 24:33–41. |
| **Ajuwon 2007**  Brieger AJA and WR. Evaluation of a school-based Reproductive Health Education Program in rural South Western, Nigeria. African J Reprod Heal. 2007. |
| **Allen 1994**  Allen JP, Kuperminc G, Philliber S, Herre K. Programmatic prevention of adolescent problem behaviors: The role of autonomy, relatedness, and volunteer service in the teen outreach program. Am J Community Psychol. 1994;22:617–38. |
| **Aplasca 1995**  Mari Rose A.Aplasca, David Siegel, Jay Paul ESH. Results of a model AIDS prevention program for high school students in the Philippines. AIDS. 1995;9. |
| **Atwood 2012**  Atwood KA, Kennedy SB, Shamblen S, Tegli J, Garber S, Fahnbulleh PW, et al. Impact of School-based HIV Prevention Program in Post-Conflict Liberia. AIDS Educ Prev. 2012;24:68–77. |
| **Austrian 2021**  Austrian K, Kangwana B, Muthengi E, Soler-Hampejsek E. Effects of sanitary pad distribution and reproductive health education on upper primary school attendance and reproductive health knowledge and attitudes in Kenya: a cluster randomized controlled trial. Reprod Health. 2021;18:1–13. |
| **Baumler 2012**  Baumler E, Glassman J, Tortolero S, Markham C, Shegog R, Peskin M, et al. Examination of the relationship between psychosocial mediators and intervention effects in It’s your game: An effective HIV/STI/pregnancy prevention intervention for middle school students. AIDS Res Treat. 2012;2012. |
| **Borawski 2009**  Elaine A. Borawski, Erika S. Trapl, Kimberly Adams-Tufts, Laura L. Hayman, Meredith A. Goodwin LDL. Taking Be Proud! Be Responsible! to the Suburbs: A Replication Study. Perspect Sex Reprod Heal. 2009;23:1–7. |
| **Borgia 2005**  Borgia P, Marinacci C, Schifano P, Perucci CA, Borgia P MCSP, Perucci CA. Is peer education the best approach for HIV prevention in schools? Findings from a randomized controlled trial. J Adolesc Heal. 2005;36:508–16. |
| **Brinkman 2016**  Brinkman SA, Johnson SE, Codde JP, Hart MB, Straton JA, Mittinty MN, et al. Efficacy of infant simulator programmes to prevent teenage pregnancy: a school-based cluster randomised controlled trial in Western Australia. Lancet. 2016;388:2264–71. |
| **Cabezón 2005**  Cabezón C, Vigil P, Rojas I, Leiva ME, Riquelme R, Aranda W, et al. Adolescent pregnancy prevention: An abstinence-centered randomized controlled intervention in a Chilean public high school. J Adolesc Heal. 2005;36:64–9. |
| **Coyle 2001***  Coyle K, Basen-Engquist K, Kirby D, Parcel G, Banspach S, Collins J, et al. Safer choices: Reducing teen pregnancy, HIV, and STDs. Public Health Rep. 2001;116 SUPPL. 1:82–93.  Coyle K, Basen-engquist K, Kirby D, Parcel G, Banspach S, Harrist R, et al. Short-Term Impact of Safer Choices : A Multicomponent School-based HIV, Other STD, and Pregnancy Prevention Program. J Sch Health. 1999;69:181–8.  Kirby DB, Baumler E, Coyle KK, Basen-Engquist K, Parcel GS, Harrist R, et al. The ``’Safer choices’’ intervention: Its impact on the sexual behaviors of different subgroups of high school students. J Adolesc Heal. 2004;35:442–52. |
| **Coyle 2021**  Coyle K, Anderson P, Laris BA, Barrett M, Unti T, Baumler E. A Group Randomized Trial Evaluating High School FLASH, a Comprehensive Sexual Health Curriculum. J Adolesc Heal. 2021;68:686–95. |
| **Cupp 2008**  Daley EM, Marhefka SL, Wang W, Noble CA, Mahony H, Arzola S, et al. Longitudinal evaluation of the Teen Outreach Programme: Impacts of a health promotion programme on risky sexual behaviours. Health Educ J. 2019;78:916–30. |
| **Daley 2019**  Daley EM, Marhefka SL, Wang W, Noble CA, Mahony H, Arzola S, et al. Longitudinal evaluation of the Teen Outreach Programme: Impacts of a health promotion programme on risky sexual behaviours. Health Educ J. 2019;78:916–30. |
| **Darabi 2017**  Darabi F, Hossein Kaveh M, Khalajabadi Farahani F, Yaseri M, MajlessiMD F, Shojaeizadeh D. Reproductive Health in Iranian Adolescent Girls: A Randomized Controlled Trial. J Res Heal Sci. 2017;17:400. |
| **Denison 2011**  Denison JA, Tsui S, Bratt J, Torpey K, Weaver MA, Kabaso M. Do peer educators make a difference? An evaluation of a youth-led HIV prevention model in Zambian Schools. Health Educ Res. 2012;27:237–47. |
| **Deveaux 2007**  Deveaux L, Stanton B, Lunn S, Cottrell L, Yu S, Brathwaite N, et al. Reduction in human immunodeficiency virus risk among youth in developing countries. Arch Pediatr Adolesc Med. 2007;161:1130–9. |
| **Eggleston 2000**  Deveaux L, Stanton B, Lunn S, Cottrell L, Yu S, Brathwaite N, et al. Reduction in human immunodeficiency virus risk among youth in developing countries. Arch Pediatr Adolesc Med. 2007;161:1130–9. |

| **Elliott 2013**  Elliott L, Henderson M, Nixon C, Wight D. Has untargeted sexual health promotion for young people reached its limit? A quasi-experimental study. J Epidemiol Community Heal. 2013;67:398–404. |
| --- |
| **Flay 2004**  Flay BR, Graumlich S, Segawa E, Burns JL, Holliday MY. Effects of 2 Prevention Programs on High-Risk Behaviors among African American Youth: A Randomized Trial. Arch Pediatr Adolesc Med. 2004;158:377–84. |
| **Fowole 1999**  Fawole IO, Asuzu MC, Oduntan SO, Brieger WR. A school-based AIDS education programme for secondary school students in Nigeria: a review of effectiveness. Health Educ Res. 1999;14:675–83. |
| **van der Maas 2009**  Van Der Maas F, Otte WM. Evaluation of HIV/AIDS secondary school peer education in rural Nigeria. Health Educ Res. 2009;24:547–57 |
| **Gelfond 2016**  Gelfond J, Dierschke N, Lowe D, Plastino K. Preventing pregnancy in high school students: Observations from a 3-year longitudinal, quasi-experimental study. Am J Public Health. 2016;106:S97–102. |
| **Goesling 2016**  Goesling B, Scott ME, Cook E. Impacts of an enhanced family health and sexuality module of the health teacher middle school curriculum: A cluster randomized trial. Am J Public Health. 2016;106:S125–31. |
| **Gómez‑Lugo 2022**  Gómez-Lugo M, Morales A, Saavedra-Roa A, Niebles-Charris J, Abello-Luque D, Marchal-Bertrand L, et al. Effects of a Sexual Risk-Reduction Intervention for Teenagers: A Cluster-Randomized Control Trial. AIDS Behav. 2022;26:2446–58. |
| **Gruchow 2011**  Gruchow HW BR. Evaluation of the Wise Guys Male Responsibility Curriculum : J Sch Health. 2011;81. |

| **Hegdahl 2022**  Hegdahl HK, Musonda P, Svanemyr J, Mumba J, Gr T, Jacobs C, et al. Effects of economic support , comprehensive sexuality education and community dialogue on sexual behaviour : Findings from a cluster-RCT among adolescent girls in rural Zambia. Soc Sci Med. 2022;306 February. |
| --- |
| **Herz 1986**  Herz EJ, Reis JS, Barbera-Stein L. Family Life Education for Young Teens: An Assessment of Three Interventions. Heal Educ Behav. 1986;13:201–21. |
| **James 2006**  James S, Reddy P, Ruiter RAC, McCauley A, Van Den Borne B. The impact of an HIV and AIDS life skills program on secondary school students in Kwazulu-Natal, South Africa. AIDS Educ Prev. 2006;18:281–94. |
| **Jemmott III 2010***  John B. Jemmott III, Loretta S. Jemmott GTF. Efficacy of a Theory-Based Abstinence-Only Intervention over 24 Months: A Randomized Controlled Trial with Young Adolescents. Arch Pediatr Adolesc Med. 2010;164(2).  Jingwen Zhang, John B. Jemmott III LSJ. Mediation and Moderation of an Efficacious Theory-Based Abstinence-Only Intervention for African American Adolescents. Physiol Behav. 2015;176:139–48. |
| **Karnell 2006**  Karnell AP, Cupp PK, Zimmerman RS, Feist-Price S, Bennie T. Efficacy of an American alcohol and HIV prevention curriculum adapted for use in South Africa: Results of a pilot study in five township schools. AIDS Educ Prev. 2006;18:295–310. |
| **Kirungi 2020**  Kirungi GK, Kiyingi FP, Kirungi GK, Kasozi J, Musoke M. Effectiveness of School Based Health Clinics on Utilization of Sexual and Reproductive Health Services Among School Girls Aged 15-19 Years in Uganda: Cluster Randomized Trial. J Clin Med Case Reports. 2020;4:1–6. |

| **Kemigisha 2019**  Kemigisha E, Bruce K, Ivanova O, Leye E, Coene G, Ruzaaza GN, et al. Evaluation of a school based comprehensive sexuality education program among very young adolescents in rural Uganda. BMC Public Health. 2019;19:1393. |
| --- |
| **Kirby 1991**  Kirby D, Barth RP, Leland N, Fetro J V. Reducing the risk: Impact of a new curriculum on sexual risk-taking. Fam Plann Perspect. 1991;23:253–63. |
| **Krugu 2018**  Krugu JK, Mevissen FEF, Van Breukelen G, Ruiter RAC. SPEEK: Effect evaluation of a Ghanaian school-based and peer-led sexual education programme. Health Educ Res. 2018;33:292–314. |
| **LaChausse 2016**  LaChausse RG. A clustered randomized controlled trial of the positive prevention PLUS adolescent pregnancy prevention program. Am J Public Health. 2016;106:S91–6. |
| **Levy 1995**  Levy SR, Perhats C, Weeks K, Handler AS, Zhu C, Flay BR. Impact of a School‐Based AIDS Prevention Program on Risk and Protective Behavior for Newly Sexually Active Students. J Sch Health. 1995;65:145–51. |
| **Lieberman 2000**  Lieberman BLD, Gray H, Wier M, Fiorentino R, Maloney P. Long-Term Outcomes of an Abstinence-Based, Small-Group Pregnancy Prevention Program In New York City Schools. Fam Plann Perspect. 2000;Volume 32,:237–45. |
| **Lohan 2022**  Lohan M, Brennan-Wilson A, Aventin Á, Gough A, Clarke M, McDowell C, et al. Effects of gender-transformative relationships and sexuality education to reduce adolescent pregnancy (the JACK trial): a cluster-randomised trial. Lancet Public Heal. 2022;7:e626–37. |
| **Manlove 2021**  Manlove J, Welti K, Whitfield B, Faccio B, Finocharo J, Ciaravino S. Impacts of Re:MIX—A School-Based Teen Pregnancy Prevention Program Incorporating Young Parent Coeducators. J Sch Health. 2021;91:915–27. |

| **Markham 2012***  Markham CM, Tortolero SR, Peskin MF, Shegog R, Thiel M, Baumler ER, et al. Sexual Risk Avoidance and Sexual Risk Reduction Interventions for Middle School Youth: A Randomized Controlled Trial. J Adolesc Heal. 2012;50:279–88.  Christine M. Markham, Melissa F. Peskin, Ross Shegog, Elizabeth R. Baumler, Robert C. Addy, Melanie Thiel, Soledad Liliana Escbar-Chaves, Leah Robin SRT. Behavioral and Psychosocial Effects of Two Middle School Sexual Health Education Programs at Tenth-Grade Follow-Up. J Adolesc Heal. 2014;176:139–48 |
| --- |
| **Mathews 2012**  Mathews C, Aaro L E Grimsrud A Flisher A J Kaaya S Onya H Schaalma H Wubs A Mukoma W Klepp K-I. Effects of the SATZ teacher-led school HIV prevention programmes on adolescent sexual behaviour: cluster randomised controlled trials in three sub-Saharan African sites. Int Health. 2012;4:111–22. |
| **Mbizvo 2023**  Mbizvo MT, Kasonda K, Muntalima N-C, Rosen JG, Inambwae S, Namukonda ES, et al. Comprehensive sexuality education linked to sexual and reproductive health services reduces early and unintended pregnancies among in-school adolescent girls in Zambia. BMC Public Health. 2023;23:348. |
| **Mellanby 1995**  Mellanby AR, Phelps F a, Crichton NJ, Tripp JH. School sex education: an experimental programme with educational and medical benefit. BMJ. 1995;311. |
| **Menna 2015**  Menna T, Ali A, Worku A. Effects of peer education intervention on HIV/AIDS related sexual behaviors of secondary school students in Addis Ababa, Ethiopia: a quasi-experimental study. Reprod Health. 2015;12:84. |
| **Michielsen 2012**  Michielsen K, Beauclair R, Delva W, Roelens K, Van Rossem R, Temmerman M. Effectiveness of a peer-led HIV prevention intervention in secondary schools in Rwanda: results from a non-randomized controlled trial. BMC Public Health. 2012;12. |

| **Millanzi 2022***  Millanzi WC, Osaki KM, Kibusi SM. The effect of educational intervention on shaping safe sexual behavior based on problem-based pedagogy in the field of sex education and reproductive health: clinical trial among adolescents in Tanzania. Heal Psychol Behav Med. 2022;10:262–90.  Millanzi WC, Kibusi SM, Osaki KM. Effect of integrated reproductive health lesson materials in a problem-based pedagogy on soft skills for safe sexual behaviour among adolescents: A schoolbased randomized controlled trial in Tanzania. PLoS One. 2022;17 2 Febuary:1–27. |
| --- |
| **Mitchell-DiCenso 1997**  Alba Mitchell-DiCenso, B. Helen Thomas, M. Corinne Devlin, Charlie H. Goldsmith, Andy Willan, Joel Singer, Susan Marks, Derek Watters SH. Evaluation of an educational program to prevent adolescent pregnancy. Heal Educ Behav. 1997;24 (3). |
| **Mmbaga 2017**  Mmbaga EJ, Kajula L, Aarø LE, Kilonzo M, Wubs AG, Eggers SM, et al. Effect of the PREPARE intervention on sexual initiation and condom use among adolescents aged 12-14: a cluster randomised controlled trial in Dar es Salaam, Tanzania. BMC Public Health. 2017;17:322. |
| **Morales 2014**  Morales A, Espada JP, Orgilés M, Secades-Villa R, Remor E. The short-term impact of peers as co-facilitators of an HIV prevention programme for adolescents: A cluster randomised controlled trial. Eur J Contracept Reprod Heal Care. 2014;19:379–91. |
| **Morales 2015**  Morales A, Espada JP, Orgilés M. A 1-year follow-up evaluation of a sexual-health education program for Spanish adolescents compared with a well-established program. Eur J Public Health. 2015;26:35–41. |
| **Morales 2020**  Morales A, Orgilés M, Espada JP, Morales A OM, Espada JP, Morales A, et al. Sexually unexperienced adolescents benefit the most from a sexual education program for adolescents: A longitudinal cluster randomized controlled study. AIDS Educ Prev. 2020;32:493–511. |
| **Mwale 2019**  Mwale M, Muula AS. The efficacy of peer education in sexual behavioral change among school-going adolescents in Northern Malawi: A quasi experiment. J HIV/AIDS Soc Serv. 2019;18:229–47.[2] |
| **Peskin 2019**  Peskin MF, Coyle KK, Anderson PM, Laris BA, Glassman JR, Franks HM, et al. Replication of It’s Your Game…Keep It Real! in Southeast Texas. J Prim Prev. 2019;40:297–323. |
| **Pinandari 2023**  Pinandari AW, H MP, Kågesten AE, Ph D, H MP, Li M, et al. Short-Term Effects of a School-Based Comprehensive Sexuality Education Intervention Among Very Young Adolescents in Three Urban Indonesian Settings : A Quasi-Experimental Study. J Adolesc Heal. 2023;73:S21–32. |
| **Piotrowski 2016**  Piotrowski ZH, Hedeker D. Evaluation of the Be the Exception Sixth-Grade Program in Rural Communities to Delay the Onset of Sexual Behavior. Am J Public Health. 2016;106:S132–9. |
| **Potter 2016**  Potter SC, Coyle KK, Glassman JR, Kershner S, Prince MS. It’s your game..keep it real in South Carolina: A group randomized trial evaluating the replication of an evidence-based adolescent pregnancy and sexually transmitted infection prevention program. Am J Public Health. 2016;106:S60–9. |
| **Ramírez-Villalobos 2021**  Ramírez-Villalobos D, Monterubio-Flores EA, Gonzalez-Vazquez TT, Molina-Rodríguez JF, Ruelas-González MG, Alcalde-Rabanal JE. Delaying sexual onset: outcome of a comprehensive sexuality education initiative for adolescents in public schools. BMC Public Health. 2021;21:1439. |
| **Rohrbach 2015**  Rohrbach LA, Berglas NF, Jerman P, Angulo-Olaiz F, Chou C-P, Constantine NA, et al. A rights-based sexuality education curriculum for adolescents: 1-year outcomes from a cluster-randomized trial. J Adolesc Heal. 2015;57:399–406. |

| **Rohrbach 2019**  Rohrbach LA, Donatello RA, Moulton BD, Afifi AA, Meyer KI, De Rosa CJ. Effectiveness Evaluation of It’s Your Game: Keep It Real, a Middle School HIV/Sexually Transmitted Infection/Pregnancy Prevention Program. J Adolesc Heal. 2019;64:382–9. |
| --- |
| **Ross 2007***  Ross DA, Changalucha J, Obasi A I, Todd J, Plummer M L, Cleophas-Mazige B, Anemona A, Everett D, Weiss H A, Mabey D C GH, Hayes RJ. Biological and behavioural impact of an adolescent sexual health intervention in Tanzania: A community-randomized trial. AIDS. 2007;21:1943–55.  Obasi AI, Cleophas B, Ross DA, Chima KL, Mmassy G, Gavyole A, et al. Rationale and design of the MEMA kwa Vijana adolescent sexual and reproductive health intervention in Mwanza Region, Tanzania. AIDS Care - Psychol Socio-Medical Asp AIDS/HIV. 2006;18:311–22  Plummer ML, Wight D, Obasi AIN, Wamoyi J, Mshana G, Todd J, et al. A process evaluation of a school-based adolescent sexual health intervention in rural Tanzania: The MEMA kwa Vijana programme. Health Educ Res. 2007;22:500–12.  Doyle AM, Weiss HA, Maganja K, Kapiga S, McCormack S, Watson-Jones D, et al. The long-term impact of the MEMA kwa Vijana adolescent sexual and reproductive health intervention: Effect of dose and time since intervention exposure. PLoS One. 2011;6:e24866. |
| **Rotz 2018**  Rotz D, Goesling B, Manlove J, Welti K, Trenholm C. Impacts of a School-Wide, Peer-Led Approach to Sexuality Education: A Matched Comparison Group Design. J Sch Health. 2018;88:549–59. |
| **Scull 2018**  Scull TM, Kupersmidt JB. Using media literacy education for adolescent sexual health promotion in middle school: Randomized control trial of Media Aware. J Heal Commun. 2018;23:1051–63. |
| **Shinde 2018*­**  Shinde S, Weiss HA, Varghese B, Khandeparkar P, Pereira B, Sharma A, et al. Promoting school climate and health outcomes with the SEHER multi-component secondary school intervention in Bihar, India: a cluster-randomised controlled trial. Lancet. 2018;392:2465–77.  Shinde S, Khandeparkar P, Bernadette S, Kendriya P, Sangathan V, Sharma A, et al. What makes multicomponent school-based health promotion interventions work? A qualitative study nested in the SEHER trial in Bihar, India. 2020;:1–21. |
| **Speizer 2020**  Ilene S. Speizer, Khou Xiong, Mahua Mandal DD. HIV-Related Knowledge, Attitudes, and Behaviors among Grade 10 Girls and Boys in Mpumalanga and KwaZulu-Natal: Cross- Sectional Results. Open AIDS J. 2020;176:139–48. |
| **Stanton 2012***  Bonita Stanton, Xinguang Chen, Veronica Koci, Lynette Deveaux, Sonja Lunn, Carole Harris, Nanika Brathwaite, Perry Gomez, Xiaoming Li and SM. Effect of a grade 6 HIV risk reduction intervention four years later among students who were and were not enrolled in the study trial. J Adolesc Heal. 2012;23:1–7.  Stanton B, Wang B, Deveaux L, Lunn S, Rolle G, Li X, et al. Assessing the effects of a complementary parent intervention and prior exposure to a preadolescent program of HIV risk reduction for mid-adolescents. Am J Public Health. 2015;105:575–83.  Bonita Stanton, Veronica Dinaj-Koci, Bo Wang, Lynette Deveaux, Sonja Lunn, Xiaoming Li, Glenda Rolle, Nanika Brathwaite, Sharon Marshall PG. Adolescent HIV risk reduction in The Bahamas: Results from two randomized controlled intervention trials spanning elementary school through high school. AIDS Behav. 2016;20(6). |
| **Stephenson 2004***  Stephenson JM, Strange V, Forrest S, Oakley A, Copas A, Allen E, et al. Pupil-led sex education in England (RIPPLE study): Cluster-randomised intervention trial. Lancet. 2004;364:338–46.  Stephenson JM, Strange V, Forrest S, Oakley A, Copas A, Allen E, et al. Pupil-led sex education in England (RIPPLE study): Cluster-randomised intervention trial. Lancet. 2004;364:338–46. |
| **Taylor 2014**  Taylor M, Jinabhai C, Dlamini S, Sathiparsad R, Eggers MS, De Vries H. Effects of a Teenage Pregnancy Prevention Program in KwaZulu-Natal, South Africa. Health Care Women Int. 2014;35:845–58. |

| **Thato 2008**  Thato R, Jenkins RA, Dusitsin N. Effects of the culturally-sensitive comprehensive sex education programme among Thai secondary school students. J Adv Nurs. 2008;62:457–69. |
| --- |
| **Tibbits 2011**  Tibbits MK, Smith EA, Caldwell LL, Flisher AJ. Impact of HealthWise South Africa on polydrug use and high-risk sexual behavior. Health Educ Res. 2011;26:653–63. |
| **Todesco 2023**  Todesco M, Breman J, Haryanto NN. Effect evaluation of a comprehensive sexuality education intervention based on socio-emotional learning among adolescents in Jakarta , Indonesia. Front Public Heal. 2023; October:1–11. |
| **Walker 2006**  Walker D, Gutierrez JP, Torres P, Bertozzi SM. HIV prevention in Mexican schools: Prospective randomised evaluation of intervention. Br Med J. 2006;332:1189–92. |
| **Wight 2002***  Daniel Wight, Gillian M Raab, Marion Henderson, Charles Abraham, Katie Buston, Graham Hart SS, Abstract. Limits of teacher delivered sex educatio:interim behavioural outcomes from randomised trial. BMJ. 2002;322 June 2002:1027–30.  Henderson M, Wight D, Raab GM, Abraham C, Parkes A, Scott S, et al. Impact of a theoretically based sex education programme (SHARE) delivered by teachers on NHS registered conceptions and terminations: Final results of cluster randomised trial. Br Med J. 2007;334:133–6. |
| **Wondimagegene 2023**  Wondimagegene YA, Debelew GT, Koricha ZB. Effectiveness of peer-led education interventions on contraceptive use, unmet need, and demand among adolescent girls in Gedeo Zone, South Ethiopia. A cluster randomized controlled trial. Glob Health Action. 2023;16. |
| **Yakubu 2019**  Yakubu I GGSRTAYMS, Yidana A, Yakubu I, Garmaroudi G, Sadeghi R, Tol A, et al. Assessing the impact of an educational intervention program on sexual abstinence based on the health belief model amongst adolescent girls in Northern Ghana, a cluster randomised control trial. Reprod Health. 2019;16:124. |

| **Zimmerman 2008**  Zimmerman RS, Cupp PK, Donohew L, Kristin Sionéan C, Feist-Price S, Helme D. Effects of a School-Based, Theory-Driven HIV and Pregnancy Prevention Curriculum. Perspect Sex Reprod Health. 2008;40:42–51. |
| --- |

***more than one report from the same study**

**Supplementary Table S5: Characteristics of included studies [ordered by study ID]**

| **Study** | |  | **Population** | | **Intervention** | | | |
| --- | --- | --- | --- | --- | --- | --- | --- | --- |
| **Author Year** | **Country** | **Study design** | **Mean age**  **(year)** | **Setting** | **No of concepts** | **Duration** | **Provider** | **Control** |
| Aarons 2000 | US | cluster RCT | 13 | high | 2 | 1 | 5 | nil |
| Abe 2016* | US | cluster RCT | 12 | middle | 3 | 2 | 1 | business-as-usual sexual health instruction |
| Aderibigbe 2008 | Nigeria | quasi-RCT | 15.63 | secondary | 1 | 2 | 5 | nil |
| Ajuwon 2007 | Nigeria | quasi-RCT | 16.5 | secondary | 4 | 3 | 2 | no intervention |
| Allen 1994 | US | quasi-RCT | 15.7 | nil | 3 | 3 | 3 | nil |
| Aplasca 1995 | Philippines | cluster RCT | 14.7 | high | 4 | 3 | 1 | Physical Education and Music (PEHM) |
| Atwood 2012 | Liberia | cluster RCT | 16 | elemantry | 2 | 2 | 5 | General Health |
| Austrian 2021 | Kenya | cluster RCT | 14.4 | primary | 2 | 3 | 5 |  |
| Baumler 2012 | US | cluster RCT | 13 | middle | 4 | 3 | 5 | regular H class |
| Borawski 2009 | US | cluster RCT | 15.1 | high | 2 | 2 | 3 | general health promotion |
| Borgia 2005 | Italy | cluster RCT | 18.3 | high | 2 | 2 | 2 | nil |
| Brinkman 2016 | Austrilia | cluster RCT | 14.8 | nil | 1 | 2 | 5 | standard health education curriculum |
| Cabezón 2005 | Chili | cluster RCT | 15.5 | high | 4 | 3 | 1 | nil |
| Coyle 2001* | US | cluster RCT | 14 | high | 3 | 1 | 1 | standard knowledge-based HIV prevention program |
| Coyle 2021 | US | cluster RCT | 15.3 | high | 6 | 4 | 5 | knowledge-based sexual health curriculum, health PE |
| Cupp 2008 | South Africa | cluster RCT | 15 | high | 1 | 4 | 2 | regular LO |
| Daley 2019 | US | cluster RCT | 14 | high | 4 | 3 | 5 | standard SH curriculum |
| Darabi 2017 | Iran | cluster RCT | 14.1 | high | 1 | 2 | 1 | no intervention |
| Denison 2011 | Zambia | quasi-RCT | 11-19+ | nil | 1 | 3 | 4 | nil |
| Deveaux 2007 | Bahamas | cluster RCT | 10.42 | elemantry | 1 | 3 | 1 | "The Wondrous Wetlands" (WW) |
| Eggleston 2000 | Jamaica | quasi-RCT | 12.2 | nil | 3 | 3 | 5 | sexuality and family life education curricula |
| Elliott 2013 | UK | quasi-RCT | 15.5 | nil | 1 | 1 | 1 | sexuality education (information and discussion) |
| Flay 2004 | US | cluster RCT | 10.8 | nil | 1 | 3 | 5 | attention-placebo health enhancement curriculum (HEC) |
| Fowole 1999 | Nigeria | quasi-RCT | 17.6 | secondary | 1 | 2 | 5 | nil |
| van der Maas 2009 | Nigeria | quasi-RCT |  | secondary | 1 | 3 | 4 | nil |
| Gelfond 2016 | US | quasi-RCT | 14.7 | high | 7 | 3 | 5 | no intervention |
| Goesling 2016 | US | cluster RCT | 12 | middle | 4 | 3 | 1 | regular school curriculum |
| Gómez‑Lugo 2022 | US | cluster RCT | 15.24 | high | 1 | 2 | 5 | no intervention |
| Gruchow 2011 | US | quasi-RCT | 11-14+ | nil | 7 | 2 | 5 | curriculum |
| Hegdahl 2022 | Zambia | cluster RCT | 14.1 | nil | 1 | 3 | 1 | standard school and health services |
| Herz 1986 | US | RCT | 13 | elementary | 3 | 4 | 5 | nil |
| James 2006 | South Africa | cluster RCT | 15.52 | secondary | 2 | 4 | 1 | odd lessons about aspects of HIV/AIDS education in a non structured format |
| Jemmott III 2010* | US | RCT | 12 | middle | 1 | 1 | 5 | health promotion control intervention |
| Karnell 2006 | South Africa | quasi-RCT | 16 | nil | 1 | 2 | 2 | regular LO |
| Kirungi 2020 | Uganda | cluster RCT | 15-19+ | secondary | 1 | 1 | 4 | no intervention |
| Kemigisha 2019 | Uganda | cluster RCT | 12.1 | primary | 2 | 1 | 5 | nil |
| Kirby 1991 | US | quasi-RCT | 15.3 | high | 1 | 3 | 1 | nil |
| Krugu 2018 | Ghana | cluster RCT | 15.84 | high | 1 | 1 | 4 | not receive any form of sexuality education |
| LaChausse 2016 | US | cluster RCT | 14.63 | high | 2 | 1 | 1 | standard health, science, or physical education curriculum |
| Levy 1995 | US | cluster RCT |  | high | 2 | 2 | 5 | basic AIDS education (current practice) |
| Lieberman 2000 | US | quasi-RCT | 12.9 | nil | 1 | 2 | 5 | nil |
| Lohan 2022 | UK | cluster RCT | 14.5 | secondary | 1 | 2 | 1 | standard SRE |
| Manlove 2021 | US | cluster RCT | 13.85 | nil | 1 | 2 | 5 | business-as-usual |
| Markham 2012* | US | cluster RCT | 12.6 | middle | 1 | 3 | 5 | regular Sexual Health Education |
| Mathews 2012 | South Africa | cluster RCT | 13 | high | 4 | 4 | 1 | no intervention |
| Mbizvo 2023 | Zambia | cluster RCT | 15 | nil | 3 | 1 | 1 | CSE and services |
| Mellanby 1995 | UK | quasi-RCT | 16 | secondary | 3 | 3 | 2* | own sexuality education program |
| Menna 2015 | Ethiopia | quasi-RCT | 16.5 | secondary | 2 | 4 | 4 | nil |
| Michielsen 2012 | Rawanda | quasi-RCT | 18.41 | secondary | 1 | 3 | 4 | HIV, STI, FP and pregnancy |
| Millanzi 2022* | Tanzania | cluster RCT | 15 | secondary | 3 | 4 | 5 | standard RH lesson LBP only |
| Mitchell-DiCenso 1997 | US | cluster RCT | 12.6 | nil | 2 | 1 | 3 | conventional sexuality education |
| Mmbaga 2017 | Tanzania | cluster RCT | 12.4 | primary | 1 | 3 | 2* | nil |
| Morales 2014 | Spain | cluster RCT | 15.72 | nil | 1 | 2 | 5 | no intervention |
| Morales 2015 | Spain | cluster RCT | 15.87 | high | 1 | 2 | 5 | no intervention |
| Morales 2020 | Spain | cluster RCT | 14.66 | high | 1 | 2 | 5 | waiting list group |
| Mwale 2019 | Malawi | quasi-RCT | 16 | secondary | 2 | 2 | 4 | health promotion package standard -as-usual life skills BCI |
| Peskin 2019 | US | cluster RCT | 13 | middle | 1 | 3 | 1 | usual care |
| Pinandari 2023 | Indonesia | quasi-RCT | 12 | high | 3 | 6 | 1 | regular SRH education |
| Piotrowski and Hedeker 2016 | US | cluster RCT | 12 | nil | 1 | 2 | 5 | no intervention |
| Potter 2016 | US | cluster RCT | 12.7 | middle | 1 | 3 | 1 | usual sexuality education |
| Ramírez-Villalobos 2021 | Maxico | cluster RCT | 13.4 | secondary | 1 | 3 | 1 | nil |
| Rohrbach 2015 | US | cluster RCT | 14.16 | high | 2 | 3 | 5 | basic sexuality education |
| Rohrbach 2019 | US | quasi-RCT | 15 | middle | 2 | 3 | 1 | nil |
| Ross 2007* | Tanzania | cluster RCT | 14-18+ | primary | 1 | 3 | 2 | no intervention |
| Rotz 2018 | US | cluster RCT | 15 | high | 2 | 1 | 4 | delay implementation |
| Scull 2018 | US | cluster RCT | 12.84 | middle | 4 | 1 | 1 | health promotion control intervention |
| Shinde 2018*­ | India | cluster RCT | 14.7 | secondary | 1 | 3 | 2 | control |
| Speizer 2020 | South Africa | cluster RCT | 13.6 | nil | 2 | 1 | 1 | non revised Life Orientation without scripted lessons plan |
| Stanton 2012* | Bahamas | cluster RCT | 14.5 | high | 1 | 1 | 1 | FYOC, "The Wondrous Wetlands" (WW) |
| Stephenson 2004* | UK | cluster RCT | 13.7 | nil | 3 | 2 | 4 | usual SRE |
| Taylor 2014 | South Africa | cluster RCT | 13.9 | high | 6 | 3 | 5 | SL program TP Program+ School Life skills Program |
| Thato 2008 | Thailand | cluster RCT | 16.67 | secondary | 4 | 2 | 5 | no intervention |
| Tibbits 2011 | South Africa | cluster RCT | 14 | nil | 2 | 3 | 1 | LO |
| Todesco 2023 | Indonesia | cluster RCT | 16-17 | secondary | 7 | 4 | 4 | no intervention |
| Walker 2006 | Mexico | cluster RCT | 18 | high | 3 | 2 | 1 | existing sexuality education course |
| Wight 2002* | UK | cluster RCT | 14 | secondary | 3 | 1 | 1 | existing sexuality education course |
| Wondimagegene 2023 | Ethiopia | cluster RCT | 16.97 | secondary | 3 | 3 | 4 | no intervention |
| Yakubu 2019 | Ghana | cluster RCT | 17-19 | high | 1 | 2 | 5 | school-delivered sexuality program |
| Zimmerman 2008 | US | quasi-RCT | 14 | high | 2 | 3 | 1 | HIV prevention, non-skill based |

RCT - Randomized Controlled Trial

cluster RCT - Cluster Randomized Controlled Trial

quasi-RCT – Quasi- Randomized Controlled Trial

**Concepts of CSE**

| 1 | relationships |
| --- | --- |
| 2 | values, rights and culture |
| 3 | gender |
| 4 | violence and staying safe |
| 5 | skills for health and well-being |
| 6 | the human body and development |
| 7 | sexuality and sexual behavior |
| 8 | sexual and reproductive health (SRH) |

**Duration**

| 1 | <12 session, 50 min each, throughout school year |
| --- | --- |
| 2 | <12 session, 50 min each, NOT throughout school year |
| 3 | >=12 session, 50 min each, throughout school year |
| 4 | >=12 session, 50 min each, NOT throughout school year |

**Provider**

| 1 | teacher |
| --- | --- |
| 2 | teacher and peer |
| 2* | teacher, peer and other |
| 3 | teacher and other |
| 4 | peer |
| 5 | other (facilitator, researcher, school health nurse) |
